# *Notch3* deletion regulates HIV-1 gene expression and systemic inflammation to ameliorate chronic kidney disease

**DOI:** 10.1242/dmm.052056

**Published:** 2025-02-25

**Authors:** Mackenzie Thornton, Nicole Sommer, Mercedes McGonigle, Anil Kumar Ram, Sireesha Yerrathota, Henrietta Ehirim, Aakriti Chaturvedi, Johnny Dinh Phan, Anubhav Chakraborty, V. Praveen Chakravarthi, Sumedha Gunewardena, Mudit Tyagi, Jaya Talreja, Tao Wang, Pravin Singhal, Pamela V. Tran, Timothy A. Fields, Patricio E. Ray, Navneet K. Dhillon, Madhulika Sharma

**Affiliations:** ^1^ Department of Internal Medicine, University of Kansas Medical Center, Kansas City, KS 66160, USA; ^2^ Department of Pathology and Laboratory Medicine, University of Kansas Medical Center, Kansas City, KS 66160, USA; ^3^ Department of Cell Biology and Physiology, University of Kansas Medical Center, Kansas City, KS 66160, USA; ^4^ Department of Medicine, Center for Translational Medicine, Thomas Jefferson University, Philadelphia, PA 19107, USA; ^5^ Division of Pulmonary, Critical Care and Sleep Medicine, Wayne State University School of Medicine and Detroit Medical Center, Detroit, MI 48201, USA; ^6^ Department of Biology, Medicine and Health, The University of Manchester, Manchester M13 9PT, UK; ^7^ Institute of Molecular Medicine, Feinstein Institute for Medical Research, Zucker School of Medicine at Hofstra-Northwell, New York, NY 11021, USA; ^8^ Child Health Research Center and Department of Pediatrics, University of Virginia School of Medicine, Charlottesville, VA 22903, USA

**Keywords:** HIV-1, Kidney, Notch3

## Abstract

Anti-retroviral therapy (ART) has decreased human immunodeficiency virus (HIV)-1-associated morbidity. However, despite ART, immune cells remain latently infected, leading to chronic inflammation and HIV-1-associated comorbidities. New strategies are needed to target viral proteins and inflammation. We found activation of Notch3 in renal cells of the HIV-1 transgenic mouse model (HIV-Tg26) and in patients with HIV-associated nephropathy. We hypothesized that targeting NOTCH3 activation constitutes an effective therapy for HIV-related chronic kidney disease. We generated HIV-Tg26 mice with *Notch3* knocked out (Tg-N3KO). Compared to HIV-Tg26 mice at 3 months, Tg-N3KO mice showed a marked reduction in renal injury, skin lesions and mortality rate. They also showed reduced renal infiltrating cells and significantly reduced expression of HIV genes. Moreover, Notch3 activated the HIV long terminal repeat promoter, and induction of HIV-1 increased Notch3 activation, indicating a feedback mechanism. Further, bone marrow-derived macrophages from HIV-Tg26 mice showed activation of Notch3, indicating systemic effects. Consistent with that observation, systemic levels of TNF and MCP-1 were reduced in Tg-N3KO compared to HIV-Tg26 mice. Thus, *Notch3* deletion/inhibition has a dual-therapeutic effect in HIV-related chronic kidney disease, which might extend to other HIV-related pathologies.

## Teaser

**Editor's choice:** This study is the first to show that Notch3 regulates HIV-1 promoter activity and reduces inflammation. Notch3 inhibition has a dual-therapeutic effect in HIV-related chronic kidney disease.

## INTRODUCTION

Anti-retroviral therapy (ART) has decreased the incidence of human immunodeficiency virus (HIV)-1-related pathologies and prolonged the lives of people with HIV-1. However, persistent low viremia exists in people with HIV-1 despite ART, which results in continuous immune activation and inflammation (Gonzales et al., 2010; Rachakonda and Kimmel, 2010; Papeta et al., 2011; Bachmann et al., 2019). These events ultimately result in chronic diseases including chronic kidney disease (CKD) (Bachmann et al., 2019; Klatt et al., 2013). Despite this, the guidelines for preventing organ damage remain the same and do not target unique molecular pathways related to disease progression (Lucas et al., 2014). We have previously reported that renal Notch signaling is activated in patients with HIV-1-associated nephropathy (HIVAN) and in non-replicating HIV-1 transgenic rodent models (Sharma et al., 2010, 2013).

Notch signaling is important for cell fate decisions in development, homeostasis and disease. Notch signaling is activated when a Notch receptor (Notch1/2/3/4) binds to a Notch ligand (Jagged or Delta) and initiates a series of proteolytic cleavages. The final cleavage is mediated by gamma secretase, which results in release of the Notch intracellular domain (NIC) and its translocation into the nucleus. In the nucleus, NIC binds to RBP-JK transcription factor and converts it into a transcriptional activator of Hes and Hey family genes (Arts and Hazuda, 2012; Dumortier et al., 2005; Giunta et al., 2011; Sharma et al., 2004). Notch signaling is essential for nephrogenesis, but its suppression is necessary for terminal differentiation of the proximal tubule cells of the kidney (Cheng et al., 2003, 2007; Cheng and Kopan, 2005; Barisoni, 2008; Bonegio et al., 2011; Waters et al., 2008; Niranjan et al., 2008). Notch overexpression has been reported in glomerular diseases including HIVAN (Niranjan et al., 2008; Bielesz et al., 2010; Murea et al., 2010; El Machhour et al., 2015; Kavvadas et al., 2018). However, most studies have focused on targeting the gamma secretase complex, which also targets other signaling pathways, including Wnt and mTOR (Efferson et al., 2010; Barat et al., 2017), and thus has not yet been successful in clinical trials. An approach to identify and target a specific disease-related Notch member holds promise. HIV-1 transgenic (HIV-Tg26) mouse and rat models are considered clinically relevant pre-clinical models to study comorbidities in people with HIV-1 on ART because these models express HIV proteins under the long terminal repeat (LTR) promoter (except Gag and Pol) but lack viral replication. These small animals exhibit disease that mimics that of patients with HIV receiving ART to reduce the effects of viral protein stress (Putatunda et al., 2018; Dickie et al., 1991; Kopp et al., 1992, 1993; Bruggeman et al., 1994; Reid et al., 2001). Targeted deletion of *Notch1* or *Notch2* is embryonic lethal; however, mice with global deletion of *Notch3* or *Notch4* develop normally (Conlon et al., 1995; Krebs et al., 2000, 2003; McCright et al., 2001). Thus, targeting Notch3 or Notch4 appears to be safe. In our previous study, global targeting of Notch4 in the HIV-Tg26 mouse model led to decreased kidney injury and increased survival (Puri et al., 2019). In the present study, we took a detailed approach to establish whether modulation of the Notch3 axis alone holds promise.

Here, we evaluated the effects of *Notch3* knockout (N3KO) in HIV-Tg26 mice. Our data show that N3KO extends the lifespan of HIV-Tg26 mice and alleviates renal pathology and function, better than *Notch4* deletion. N3KO led to a marked reduction in renal infiltrating cells and decreased the expression of HIV-1 genes in the kidneys. Bone marrow-derived macrophages (BMDMs) from HIV-Tg26 mice had activated Notch3. Finally, N3KO led to systemic reduction of inflammatory markers, which could be an underlying mechanism for improved disease and skin lesions, and prolonged life expectancy. Thus, Notch3 inhibition offers a dual-protective mechanism in HIV-related inflammation.

## RESULTS

### Notch3 activation in HIV-positive human and mouse kidneys

**Fig. 1.**
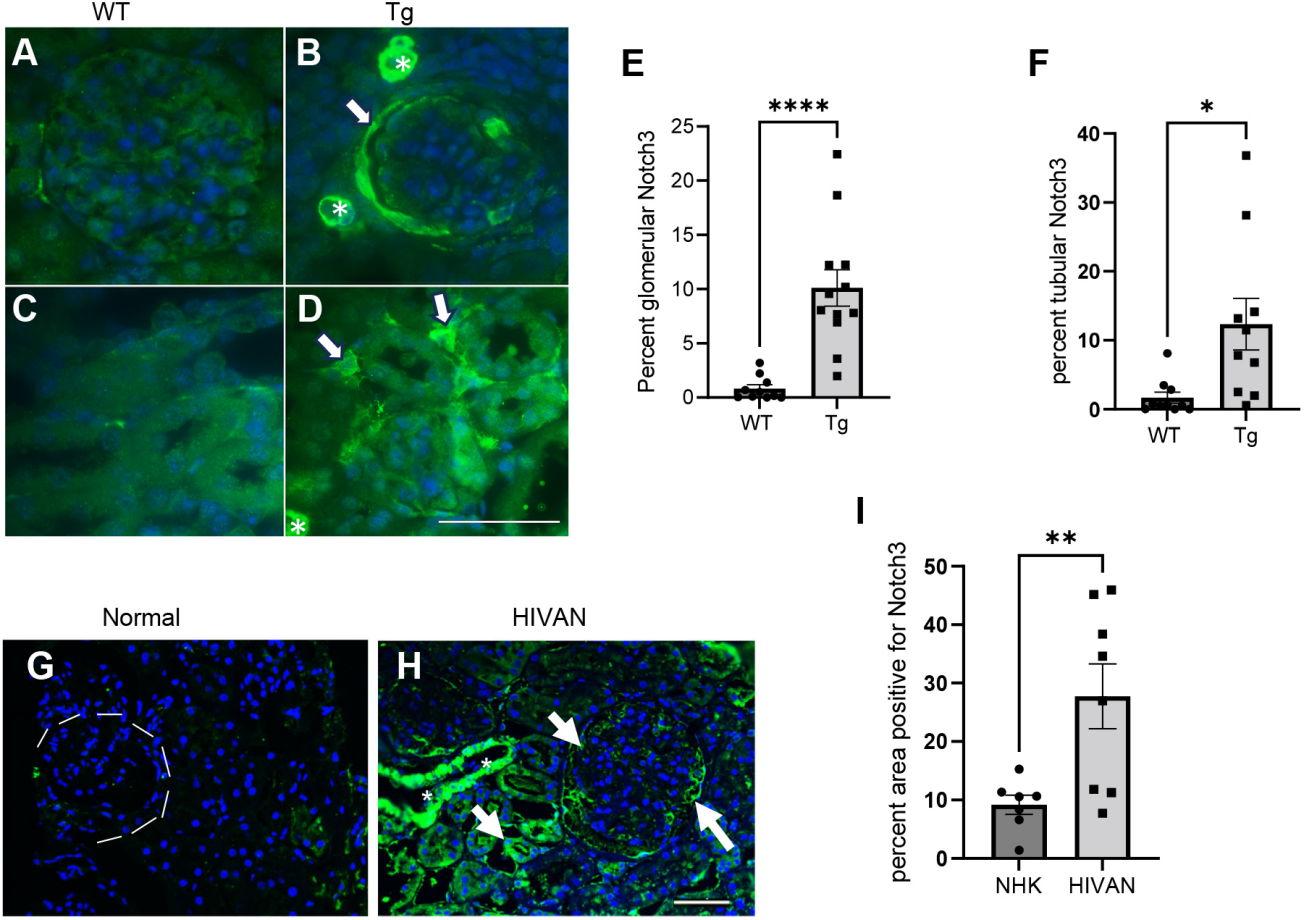
**Activation of Notch3 in HIV-1-associated nephropathy.** (A-D) Immunolabeling was performed for the presence of Notch3 in renal paraffin sections. Notch3 (green) labeling in kidney sections of 3-month-old wild-type (WT) (A,C) and HIV-Tg26 (Tg) (B,D) mice is shown. Arrows indicate glomerular and tubular interstitial cells highly positive for Notch3 expression. Asterisks indicate blood vessels in which Notch3 is normally expressed. (E,F) Quantification of glomerular (E) and tubular (F) Notch3 expression (intensity, green) as assessed using ImageJ. (G,H) Kidney biopsy from an unaffected individual (G) versus kidney biopsy from a patient with HIV-1-associated nephropathy (HIVAN) (H) (representative of *n*=3 in each group), showing nuclear NOTCH3 expression (arrows), indicating activation. Asterisks indicate blood vessels with bright Notch3 expression. Dashed line outlines glomerulus with no Notch3 expression. (I) Quantification of NOTCH3 expression (intensity, green) in patients with HIVAN and unaffected individuals (NHK), as assessed using ImageJ. Unpaired two-tailed Student's *t*-test was used; data are presented as percentage area positive for green labeling. All experiments were performed in triplicates. *P<0.05, **P<0.01, ****P<0.0001. Scale bars: 50 µm.

The renal expression of Notch3 was evaluated in HIV-Tg26 mice and compared to that in age-matched wild-type (WT) mice. Notch3 is normally expressed in vascular smooth muscle cells and was seen expressed in both WT and HIV-Tg26 renal sections (Fig. 1A,B, asterisks). However, Notch3 can be expressed in parietal epithelial cells, podocytes and tubular cells upon injury (Murea et al., 2010; El Machhour et al., 2015; Kavvadas et al., 2018; Idowu et al., 2018). Consistent with this, we found that Notch3 was brightly labeled in the parietal epithelial cells lining the Bowman capsule and in glomerular cells (Fig. 1B, arrow) in HIV-Tg26 kidney sections compared to WT kidney sections. In tubular regions, Notch3 was elevated in epithelial and interstitial cells (Fig. 1C,D, arrows). Quantitative analysis revealed a significant increase in Notch3 expression in glomerular and tubular regions in HIV-Tg26 mice compared to that in WT mice (Fig. 1E,F). Further, in patients diagnosed with HIVAN, we found that NOTCH3 was expressed and elevated in glomerular, tubular and interstitial cells (Fig. 1G,H, arrows). Quantification of renal NOTCH3 staining showed a significant increase in biopsies from patients with HIVAN compared to that in biopsies from unaffected individuals (Fig. 1I).

### Notch3 inhibition improves renal function and increases the lifespan of HIV-Tg26 mice

**Fig. 2.**
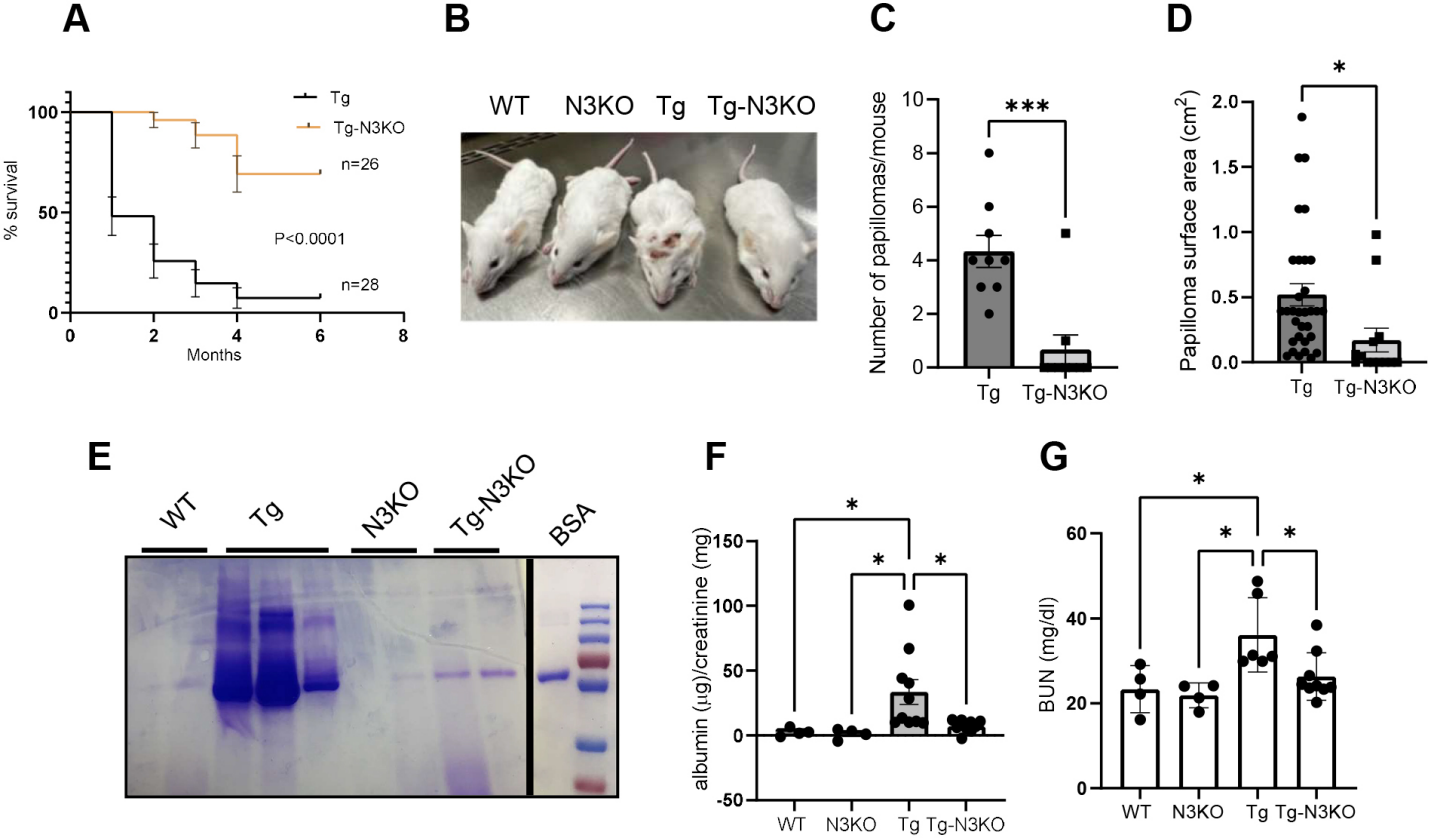
***Notch3* deletion improves disease progression and lifespan in HIV-Tg26 mice.** (A) Kaplan–Meier curve showing the 6 months mortality rate in HIV-Tg26 (Tg) mice (*n*=26) and HIV-Tg26 with *Notch3* knocked out (Tg-N3KO) mice (*n*=28). (B) Phenotypic appearance of WT, WT with *Notch3* knocked out (N3KO), Tg and Tg-N3KO mice; note the skin papillomata on the forehead of the Tg mouse, whereas the Tg-N3KO mouse appears normal. (C,D) Skin lesions/mouse were quantified (*n*=9 each), and area per lesion was measured and expressed as surface area per cm^2^. Unpaired two-tailed Student's *t*-test was used for statistical analysis. (E) Urine was collected in metabolic cages overnight from WT, N3KO, Tg and Tg-N3KO mice at 3 months of age before euthanasia. Proteinuria was assessed in 2 µl urine by SDS-PAGE followed by Coomassie Blue staining of the gels. Bovine serum albumin (BSA) was used as a positive control. (F) Albumin and creatinine ratio in urine was measured using ELISA (*n*=4, WT; *n*=4, N3KO; *n*=10, Tg; *n*=10, Tg-N3KO) and expressed as µg albumin/mg creatinine. One-way ANOVA. (G) Renal function was also assessed in serum using a blood urea nitrogen (BUN) assay kit, and results are expressed as BUN mg/dl (*n*=4, WT; *n*=4, N3KO; *n*=6, Tg; *n*=7,Tg-N3KO). One-way ANOVA. *P<0.05, ***P<0.001.

Notch3 expression was not restricted to one cell type; thus, we generated HIV-Tg26 mice with global deletion of *Notch3* (HIV-Tg-N3KO) to determine the *in vivo* downstream function of Notch3 activation. HIV-Tg-N3KO mice were born in the normal Mendelian fashion and were active and fertile. By 6 months of age, ∼10% of HIV-Tg26 mice (*n*=28) had survived, whereas 75% of HIV-Tg-N3KO mice (*n*=26) had survived (Fig. 2A). HIV-Tg26 mice presented with skin papillomata and ulcers by the age of 3 months; these abnormalities were strikingly low in HIV-Tg-N3KO mice, and quantification indicated a significant decrease in the numbers and sizes of skin papillomata/ulcers in HIV-Tg-N3KO mice compared to those in HIV-Tg26 mice (Fig. 2B-D). HIV-Tg-N3KO mice had a significant improvement in proteinuria as assessed by SDS-PAGE, and the urinary albumin and protein creatinine ratio was markedly lower in HIV-Tg-N3KO mice than that in HIV-Tg26 mice (Fig. 2E,F). Further, blood urea nitrogen (BUN) levels in HIV-Tg-N3KO mice were decreased compared to those in the HIV-Tg26 mice, indicating an overall improvement in renal function (Fig. 2G).

### *Notch3* deletion ameliorates renal injury in HIV-Tg26 mice

**Fig. 3.**
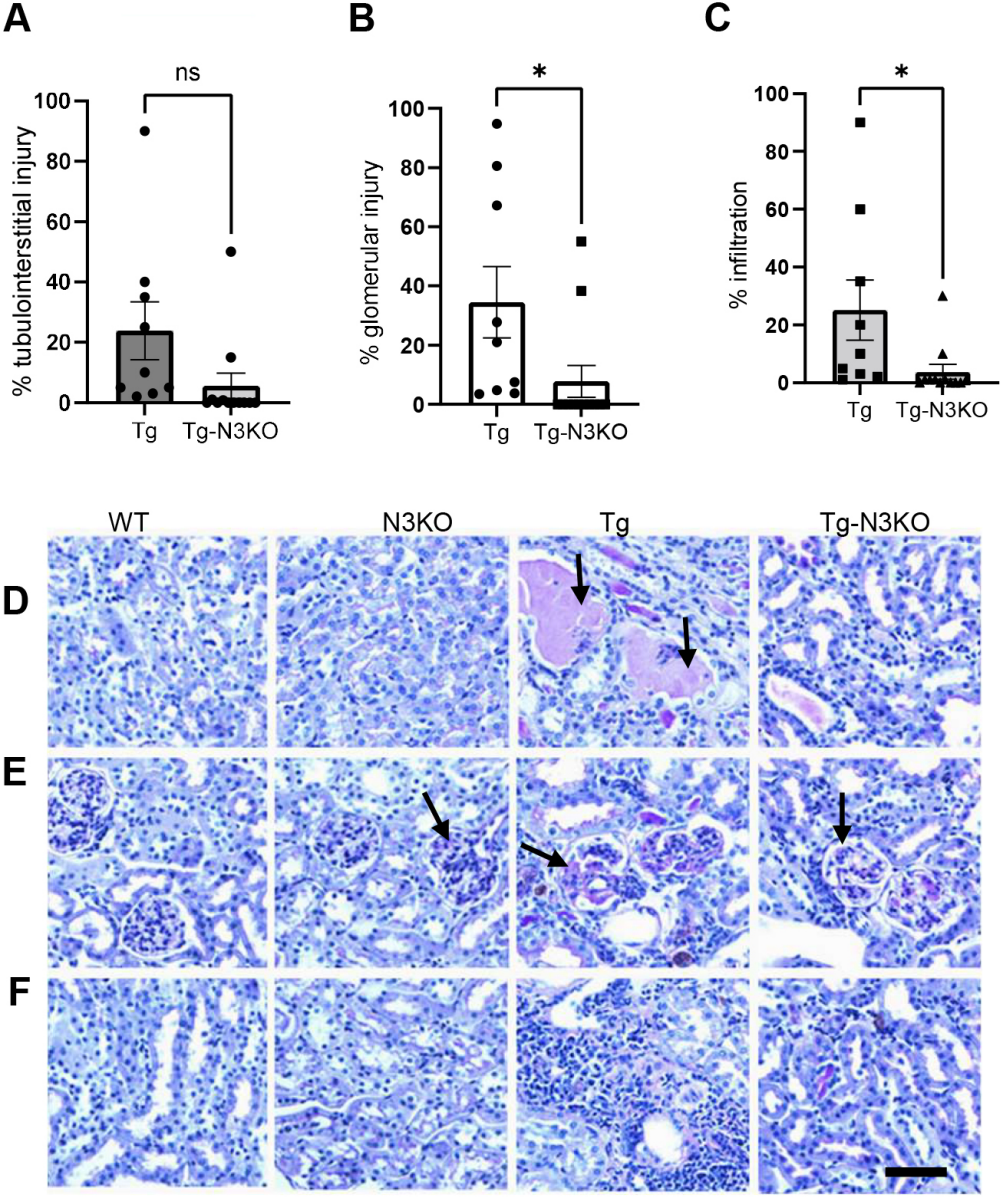
***Notch3* deletion ameliorates kidney injury in HIV-Tg26 mice.** Kidney sections from 3-month-old WT, N3KO, Tg and Tg-N3KO mice were stained with periodic acid–Schiff (PAS) to determine kidney injury. (D-F) PAS staining indicated tubulointerstitial injury (D), glomerular tubular injury (E) and inflammation (F). Note the severe kidney injury in Tg kidneys (*n*=9) compared to Tg-N3KO kidneys (*n*=12). Arrows in D indicate protein casts; arrows in E indicate comparisons between normal-looking (N3KO), Tg26 and Tg-N3KO glomeruli, showing the extent of sclerosis. (A-C) Quantitation of percentage tubulointerstitial injury (A), percentage glomerular injury (B) and percentage infiltration (C). Unpaired two-tailed Student's *t*-test was used for statistical analysis. ns, not significant; *P<0.05. Scale bar: 100 µm.

To detect histological changes in kidneys conferred by N3KO, tubulointerstitial injury, glomerular injury and infiltrating immune cells were quantified. The N3KO mice (WT mice with *Notch3* knocked out) did not show any phenotypic or histological abnormalities and were comparable to WT mice. In contrast, HIV-Tg26 mice showed tubular injury, which was reduced in HIV-Tg26-N3KO mice, although these changes did not reach statistical significance (Fig. 3A,D). Glomerular injury in HIV-Tg-N3KO mice was significantly reduced compared to that in HIV-Tg26 mice (Fig. 3B,E). Immune cell infiltration was also markedly reduced in HIV-Tg-N3KO mice compared to that in HIV-Tg26 mice (Fig. 3C,F). In addition, fibrotic lesions, as assessed by Mason trichrome staining, were highly positive in HIV-Tg26 kidneys compared to HIV-Tg-N3KO kidneys. As expected, kidneys from both WT and N3KO mice showed no fibrotic lesions (Fig. S1).

### *Notch3* deletion inhibits HIV-LTR activity

**Fig. 4.**
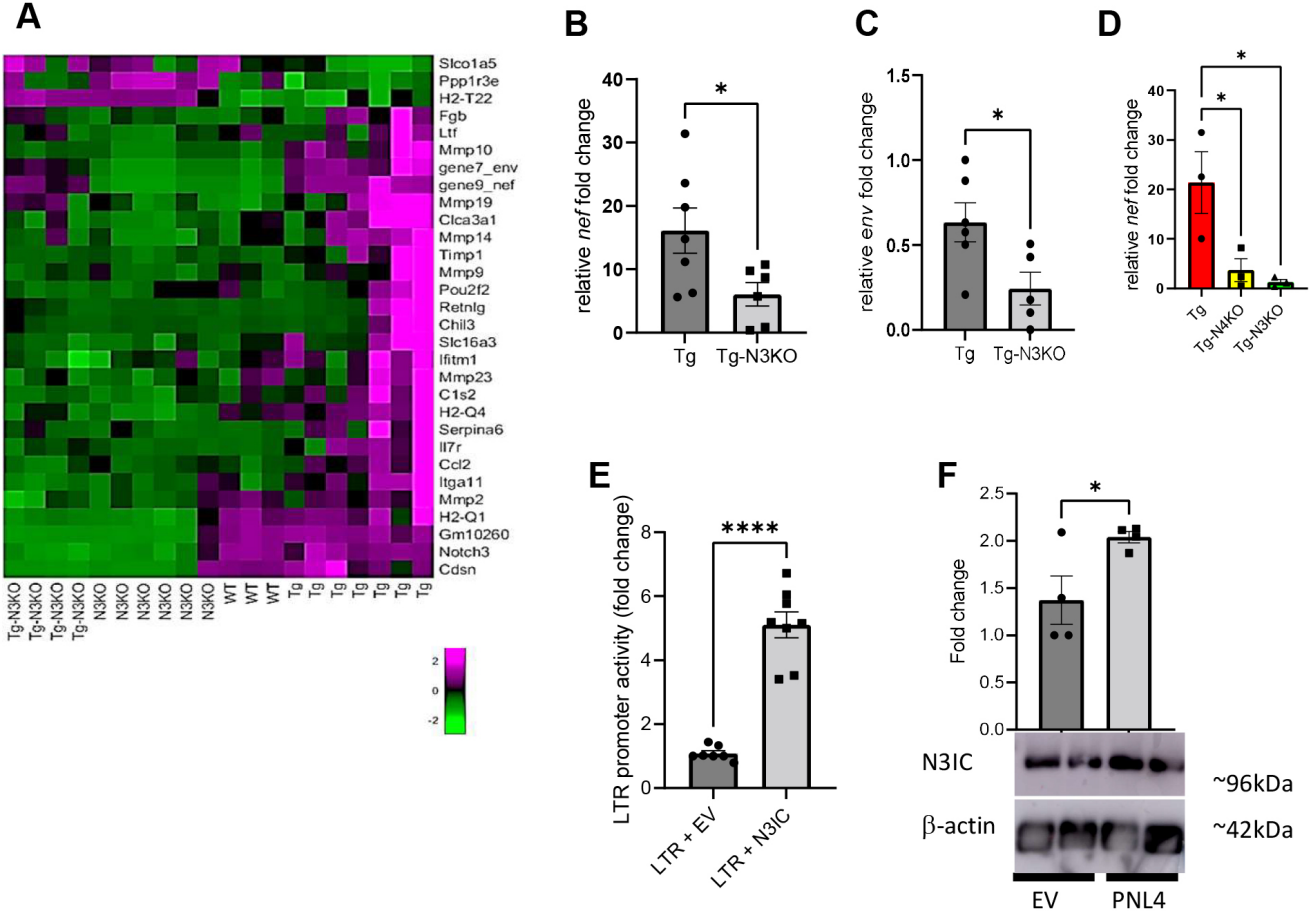
**Notch3 targets HIV-1 activity.** (A) Heatmap showing differential expression of genes obtained from mRNA sequencing of kidneys from 3-month-old WT, N3KO, Tg and Tg-N3KO mice. Note the asterisks for *nef* and *env*. (B,C) Quantitative PCR (qPCR) validating *nef* (B) and *env* (C) (*n*=5-7), followed by unpaired two-tailed Student's *t*-test for statistical analysis. (D) qPCR to compare Tg-N3KO and Tg-N4KO in repressing *nef* (*n*=3 per condition). One-way ANOVA followed by Tukey's test was used for statistical analysis. (E) HIV-long terminal repeat (LTR) promoter luciferase construct and Notch3 intracellular domain (N3IC) or PCDNA3.1 [empty vector (EV)] were transiently transfected into podocytes, followed by promoter reporter luciferase assays. Data were averaged from eight assays in three independent experiments. Vector-transfected control in each experiment was set to one, and relative fold change in LTR promoter activity was calculated. (F) Podocytes were transfected with HIV-LTR construct (PNL4) or EV. After 24 h, lysates were subjected to western blot analysis of N3IC and β-actin. Data were normalized to β-actin via ImageJ and presented as fold change (*n*=4). Unpaired two-tailed Student's *t*-test was used for statistical analysis. *P<0.05, ****P<0.0001.

To determine downstream targets of Notch3, RNA sequencing (RNA-seq) was conducted on kidneys of WT, N3KO, HIV-Tg26 and HIV-Tg-N3KO mice. Transcriptome analysis revealed many inflammatory marker genes among the most upregulated genes in HIV-Tg26 mice (Table 1). Strikingly, we also saw that the expression of HIV genes *nef* and *env* was downregulated in HIV-Tg-N3KO mice compared to that in HIV-Tg26 mice (Fig. 4A, asterisks; Table 1). These results were confirmed by quantitative PCR (qPCR), whereby a twofold decrease in *nef* and *env* mRNA expression was apparent in HIV-Tg-N3KO mice compared to that in HIV-Tg26 mice (Fig. 4B,C). Previously, we reported that *Notch4* knockout (N4KO) in HIV-Tg26 mice also ameliorates disease by reducing inflammation (Puri et al., 2019). Thus, to identify differences in the expression of *nef* and *env* upon N3KO and N4KO in HIV-Tg26 mice, we compared the transcriptomic data from HIV-Tg-N3KO mice with those from HIV-Tg26 mice with global deletion of *Notch4* (HIV-Tg-N4KO) (Table 1). Both HIV-Tg-N3KO and HIV-Tg-N4KO mice had lower *env* and *nef* expression than did HIV-Tg26 mice. qPCR further confirmed the results for *nef* (Fig. 4D). To determine whether the effects on HIV genes are directly related to activation of the HIV-LTR promoter by Notch3 intracellular domain (N3IC), we conducted promoter reporter assays in podocytes. The N3IC construct was able to increase the LTR promoter activity fivefold, indicating that *nef* and *env* reduction by N3KO was a result of direct effect on the HIV-LTR promoter (Fig. 4E). In reverse experiments, we infected a human podocyte cell line with either pseudotyped HIV-1 (PNL4) lentiviral vector or its corresponding empty vector. Western blot analysis of lysates from these cells demonstrated significant upregulation of N3IC (Fig. 4F), revealing a feedback mechanism.

**Table 1.** Genes downregulated in Tg-N3KO and Tg-N4KO mice compared to Tg26 mice

	Tg-N3KO versus Tg26		Tg-N4KO versus Tg26	
Gene ID	FC	*P*-value	FC	*P*-value
*Chil3*	−133.05	0.000931	−77.2327	0.000114
*S100a9*	−17.8874	7.51×10^−5^	−7.92142	0.000967
*Mmp10*	−14.5953	0.001577*	−1.90802	0.281182
*Il7r*	−9.05227	5.90×10^−5^	−2.62872	0.04056
*Ccl2*	−14.9554	1.44×10^−5^*	−1.4202	0.459143
*Retnlg*	−11.5474	0.014857	−12.7715	0.004266
*Itgam*	−4.48867	0.000205	−2.31992	0.012634
*nef*	−2.53002	0.00411	−1.79754	0.035797
*env*	−1.64505	0.025401	−1.85543	0.001879

Genes that were most upregulated genes in Tg26 versus wild-type mice were selected, and their expression was compared with that in Tg-N3KO or Tg-N4KO mice. Note that many genes were downregulated significantly, as shown by the *P*-values for both Tg-N3KO and Tg-N4KO mice, except for *Ccl2* and *Mmp10*, which were significantly downregulated in Tg-N3KO mice but not in Tg-N4KO mice (asterisks). FC, fold change; Tg26, HIV-1 transgenic mice; Tg-N3KO, HIV-Tg26 mice with *Notch3* knocked out; Tg-N4KO, HIV-Tg26 mice with *Notch4* knocked out.

### *Notch3* deletion inhibits the invasion of inflammatory cells in kidneys from Tg26 mice

**Fig. 5.**
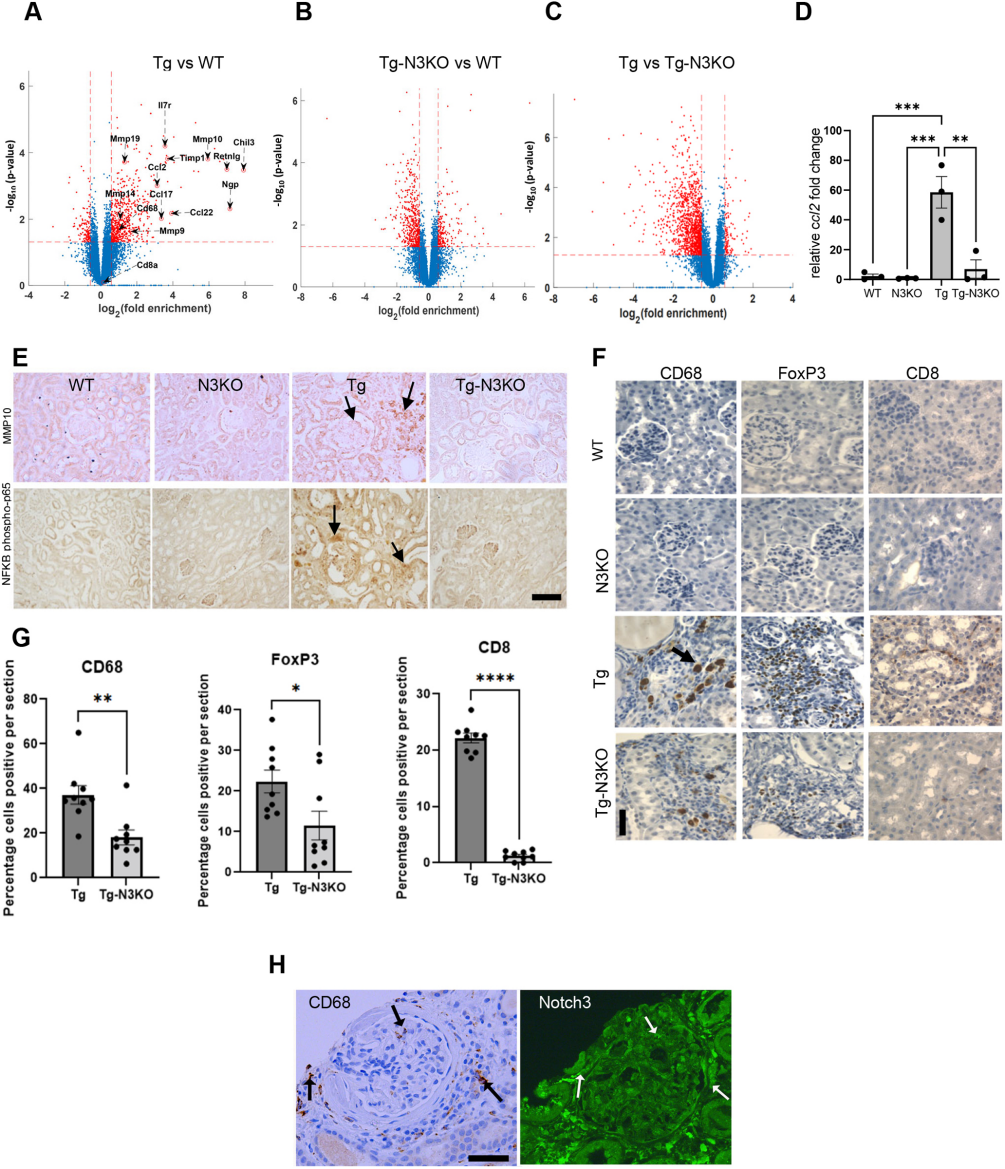
**Notch3 targets immune cell infiltration.** (A-C) Volcano plots comparing gene expression in kidneys from Tg versus WT (A), Tg-N3KO versus WT (B), and Tg versus TG-N3KO (C) mice. (D) qPCR for *Ccl2* expression. One-way ANOVA followed by Tukey's test was used for statistical analysis. (E) Immunohistochemistry (IHC) for MMP10 and NFκB (p65) in renal sections of WT, N3KO, Tg and Tg-N3KO mice. Arrows show glomerular and tubular areas with high expression. Scale bar: 100 µm. (F) Paraffin sections from kidneys of WT, N3KO, Tg and Tg-N3KO mice, labeled for immune cell markers CD68, FOXP3 and CD8. Arrow indicates CD68^+^ macrophages found in clumps in Tg kidney. Scale bar: 50 µm. (G) Quantification of CD68^+^, FOXP3^+^ and CD8^+^ HIVAN cells from Tg and Tg-N3KO mice, assessed in Tg (*n*=9) and Tg-N3KO (*n*=9) kidneys unaware of experimental group. Each dot represents the percentage of positive cells from the entire kidney where inflammatory invasions were prominent. Unpaired two-tailed Student's *t*-test was used for statistical analysis. (H) Serial sections from a kidney biopsy from a patient with HIVAN immunostained for the presence of CD68 and NOTCH3. Note the presence of CD68^+^ cells in and around glomerulus; the same areas were also labeled brightly for NOTCH3 (arrows). Scale bar: 100 µm. *P<0.05, **P<0.01, ***P<0.001, ****P<0.0001.

Volcano plots derived from RNA-seq analysis indicated that differentially expressed genes in HIV-Tg26 versus WT kidneys – such as chitinase-like 3 (*Chil3*), resistin like gamma (*Retnlg*), chemokine (C-C motif) ligand 2 (*Ccl2*) and matrix metallopeptidase 10 (*Mmp10*) – skewed towards highly upregulated [Log_2_ (fold enrichment)] in HIV-Tg26 kidneys (Fig. 5A). These genes were undifferentiated between Tg-N3KO and WT mouse kidneys and skewed towards downregulated (Fig. 5B,C). We have previously reported a list of differentially expressed genes between HIV-Tg26 and WT mice (Chakravarthi et al., 2020). Genes that were most significantly downregulated in HIV-Tg-N3KO versus HIV-Tg26 kidneys are shown in Table 1. Raw data were submitted to the Sequence Read Archive (SRA) website with accession numbers PRJNA578136 (WT and HIV-Tg26), PRJNA680191 (N3KO), PRJNA1010236 (HIV-Tg-N3KO) and PRJNA580295 (N4KO and HIV-Tg-N4KO).

Previously, we reported that N4KO in HIV-Tg26 mice also ameliorates disease by reducing inflammation (Puri et al., 2019). Thus, to identify differences between N3KO and N4KO in HIV-Tg26 mice, we compared the transcriptomic data from HIV-Tg-N3KO mice with those from HIV-Tg-N4KO mice (Table 1). Compared to HIV-Tg-N4KO mice, HIV-Tg-N3KO mice were more protected against disease, as indicated by the robust decrease in the expression of certain inflammatory genes, such as *Mmp10* and *Ccl2* (Mora-Gutiérrez et al., 2020; Wang et al., 2022; Xiromerisiou et al., 2022; Juchniewicz et al., 2017; Aqrawi et al., 2018). In HIV-Tg26 kidneys, we confirmed the upregulation of *Ccl2* via qPCR, and that of MMP10 via immunohistochemistry, and found upregulated NFκB (p65; also known as RELA) and MMP10 expression, indicating inflammatory activity (Fig. 5D,E). Further, we validated the upregulation of other inflammatory genes known to play a major role in HIV pathogenesis (*Tnf*, *S100a9*, *Itgam*) (Fig. S2). Together, these data indicate that N3KO alleviates HIV activity and associated inflammation in the kidneys of HIV-1 mice. Next, we investigated whether N3KO leads to an overall reduction in the number of CD68^+^ macrophages. CD68^+^ macrophages were found in clumps in HIV-Tg26 kidneys (Fig. 5F, arrow). Quantification of these cells revealed a 50% reduction in HIV-Tg-N3KO kidneys compared to HIV-Tg26 kidneys (Fig. 5G). In contrast, WT and N3KO kidney sections were negative for CD68 (Fig. 5E). To assess other infiltrating cells that N3KO might affect, we conducted staining for FOXP3 (a marker of regulatory T cells), CD8 (found in T lymphocytes), granzyme B (found in natural killer cells and cytotoxic T cells) and CD11c (also known as ITGAX; a marker of dendritic cells). Interestingly, there was a marked increase in the FOXP3^+^ and CD8^+^ (Fig. 5F,G) cells; however, granzyme B^+^ or CD11c^+^ cells were not found in kidneys (Fig. S3). These data indicate that N3KO reduced the invasion of many inflammatory cell types in the HIV-Tg26 kidneys. We also found CD68^+^ cells in extraglomerular areas as well as inside glomeruli in biopsies from patients with HIVAN (Fig. 5H). Notably these areas/cells showed increased NOTCH3 expression (Fig. 5H, right), indicating that CD68^+^ macrophages are rich in NOTCH3.

### Notch3 activation in BMDMs and deletion of *Notch3* alleviate systemic inflammation

**Fig. 6.**
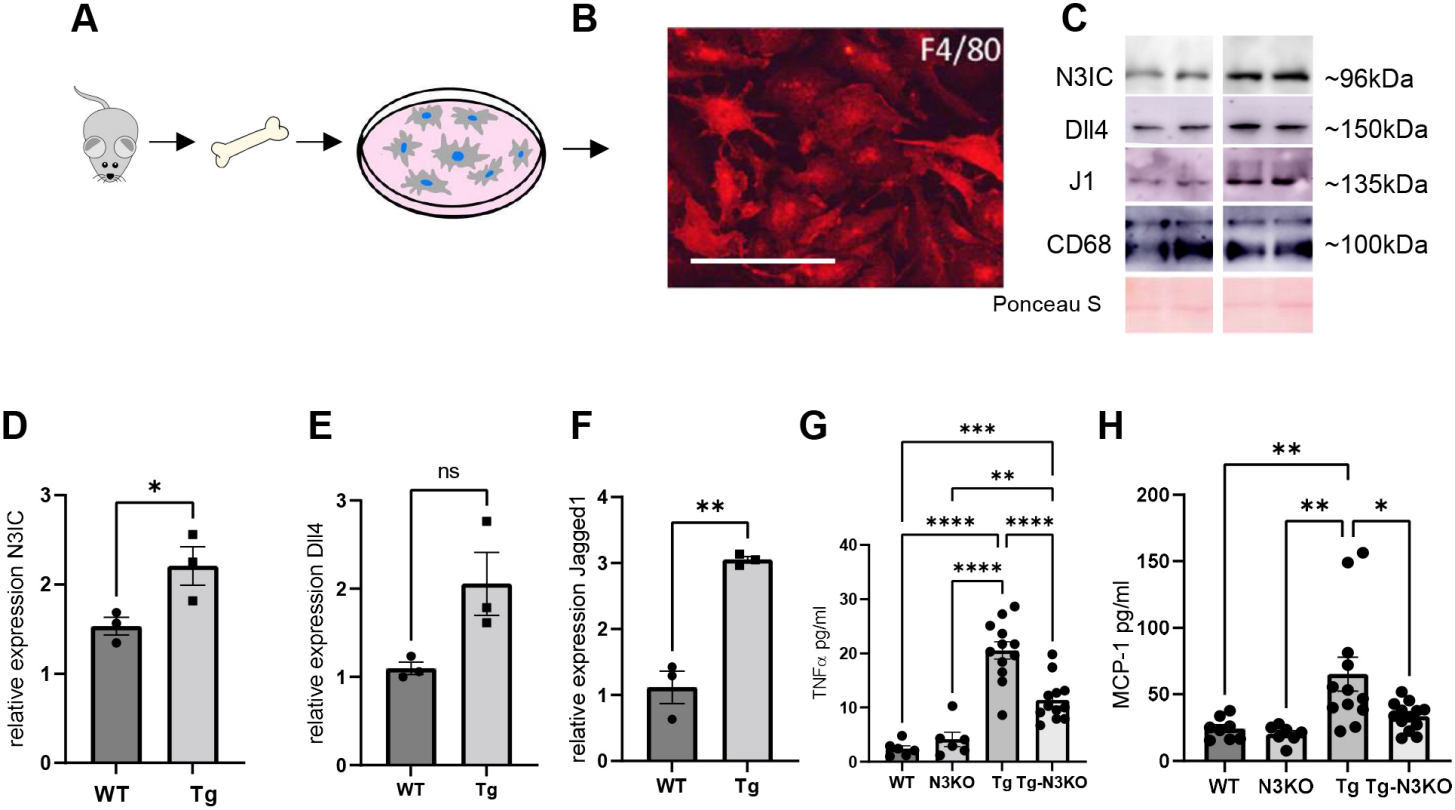
***Notch3*** **deletion targets systemic inflammation in HIV-Tg26 mice.** (A) Schematic showing macrophage isolation from bone marrow in mice. (B) Labeling of differentiated bone marrow cells expressing the macrophage marker F4/F80 in fixed cells. Scale bar: 50 µm. (C) Lysates obtained from differentiated macrophages of WT and Tg mice were subjected to western blot analysis for N3IC, Dll4 and jagged 1 (J1). CD68 was used as a loading control. Ponceau S provides an additional loading control. (D-F) Quantification of N3IC (D), Dll4 (E) and jagged 1 (F) in bone marrow cells obtained from three mice in each group (*n*=3). Unpaired two-tailed Student's *t*-test was used for statistical analysis. (G,H) ELISA was performed for the presence of TNFα (TNF; G) and MCP-1 (H) in serum obtained from 3-month-old WT, N3KO, Tg and Tg-N3KO mice (*n*=6-12), both males and females. Data are expressed in pg/ml. One-way ANOVA followed by Tukey's test was used for statistical analysis. ns, not significant; *P<0.05, **P<0.01, ***P<0.001, ****P<0.0001.

Because macrophage markers and infiltrating cells were reduced in HIV-Tg-N3KO kidneys, we reasoned that recruitment of mononuclear cells/macrophages from the bone marrow play a role in the pathogenesis of renal and systemic lesions in HIV-Tg26 mice. To determine whether BMDMs from HIV-Tg26 mice have active Notch signaling compared to those derived from WT mice, we isolated BMDMs. Cells stained positive for F4/80 (also known as ADGRE1) at differentiation (Fig. 6A,B). Western blots revealed that renal N3IC and jagged 1 expression was significantly increased in HIV-Tg26 mice compared to that in WT controls (Fig. 6C-F); although delta like 4 (Dll4) expression was observed to increase in HIV-Tg26 mice compared to that in WT controls, this did not reach significance. These data reflect that Notch pathway activation in the BMDMs itself could be responsible for inflammation in the kidneys and other organs of HIV-Tg26 mice. Next, we measured the circulating levels of TNF (formerly known as TNFα) and MCP-1 (encoded by *Ccl2*) to determine whether BMDM activation of Notch3 relates to overall inflammation**.** Our data indicated that HIV-Tg-N3KO mice had a significant reduction in the levels of serum TNF and MCP-1 compared to those in HIV-Tg26 mice (Fig. 6G,H). In addition, dot blot analysis indicated reduction in the levels of various cytokines and chemokines in the serum of HIV-Tg-N3KO mice compared with that of HIV-Tg26 mice (Fig. S4, Tables S1 and S2). Collectively, these data suggest that dysregulation in the Notch pathway of bone marrow cells in HIV-Tg26 mice translated to systemic inflammation and, thus, infiltration of immune cells into various tissues. *Notch3* deletion ameliorated these defects, and the overall phenotype of the HIV-Tg-N3KO mice was improved relative to that of HIV-Tg26 mice, including marked improvement in skin lesions.

## DISCUSSION

Our study describes HIV-1 gene regulation and inflammation as a novel dual mechanism by which the Notch3 pathway affects the outcomes and survival of HIV-Tg26 mice. Furthermore, to the best of our knowledge, this study demonstrates, for the first time, that inhibition of Notch3 signaling can specifically improve outcome from HIV-related CKD, uncovering a potential new target to prevent CKD progression in people with HIV-1.

ART is a clinically successful cocktail of anti-viral drugs (albeit with side effects) that target various stages of the HIV replication cycle. HIV-Tg26 mice carry a 7.4 kb proviral HIV-1 DNA construct containing a deletion encompassing most of the *gag* and *pol* genes, and thus HIV-1 cannot replicate in these mice. Regardless of the absence of *gag* and *pol*, the HIV-LTR promoter is fully functional to support the expression of other HIV genes (e.g. *nef*, *env*, *vpr*) and hence proteins in the HIV-Tg26 mice and cause several lesions, including skin papillomata, cataracts and CKD (Putatunda et al., 2018; Kopp et al., 1993; Cheung et al., 2015). The kidney disease in this model histologically mimics HIV-associated nephropathy in humans, which can be studied for its effects in the absence of ART owing to absence of active viral replication. However, this model is clinically relevant to ART-treated patients with HIV, as they lack active viral replication but experience continuous stress from viral proteins. Thus, HIV-Tg26 mice, although not perfect, are useful to ask specific questions related to HIVAN and HIV-CKD.

Using the HIV-1 rodent models, we have previously shown that Notch ligands and receptors are activated in a variety of renal cells (Sharma et al., 2010, 2013). In previous studies, *Notch4* deletion in HIV-Tg26 mice resulted in marked improvement in renal function, increase in lifespan and reduction in renal inflammatory infiltrates. However, the Notch3 expression pattern and its role were not clearly defined (Sharma et al., 2013). Here, we show that Notch3 was expressed and elevated in both glomerular and extraglomerular regions in HIV-Tg26 mice. A marked increase in Notch3 expression in cells lining the Bowman capsule was observed compared to that in WT mice. This might signify the regeneration potential of PEC progenitors into podocytes, and the role of Notch in this phenomenon has been studied in many other kidney injury models (El Machhour et al., 2015; Lasagni et al., 2010; Breitkopf et al., 2020; Roeder et al., 2015; Kaverina et al., 2020). This area in HIV-associated kidney diseases remains a topic for future investigations. N3KO resulted in amelioration of renal disease progression and increased lifespan in HIV-Tg26 mice. Compared to N4KO, N3KO resulted in better survival rate of HIV-Tg26 mice by 6 months (75% compared to 65%). RNA-seq data showed macrophage-associated inflammatory genes to be upregulated in HIV-Tg26 kidneys, which normalized upon N3KO compared to N4KO. MMP family genes were upregulated in HIV-Tg26 kidneys; however, *Mmp10* and *Ccl2* were uniquely downregulated upon N3KO compared to N4KO. The MMP proteins either promote or restrict macrophage flux into the tissues (Hautamaki et al., 1997). In the kidneys of patients with diabetes, MMP10 contributed to the inflammatory macrophage response, which was ameliorated by MMP10 knockout (Toni et al., 2013). The Notch target protein Hey2 has been reported to regulate MMP10 (Wu et al., 2011). We show that protein expression of MMP10, similar to NFκB (p65), was reduced by N3KO.

The role of Notch3 in inflammation has been studied in non-HIV kidney diseases. Notch3 was activated in lupus nephritis and extra capillary glomerulonephropathy (Kavvadas et al., 2018; El Machhour et al., 2015). In nephrotoxic sheep serum (NTS)-induced kidney injury and ischemia reperfusion injury, N3KO mice exhibited disease protection compared to WT mice; renal macrophages derived from N3KO mice failed to activate inflammatory cytokines (Kavvadas et al., 2018). *In vitro*, overexpression of active Notch3 in podocytes led to a reorganized cytoskeleton, leading to a migratory, proliferative and proinflammatory phenotype (El Machhour et al., 2015). We observed less inflammatory infiltration in HIV-Tg-N3KO mice than that in HIV-Tg26 mice, reasoning that this might be due to fewer infiltrating cells from extrarenal sources. The RNA-seq results supported our reasoning, showing a marked decrease in macrophage-associated markers in HIV-Tg-N3KO kidneys compared to those in HIV-Tg26 kidneys. Thus, we focused on the BMDMs. To our surprise, we found activation of Notch3 signaling and upregulation of Notch ligands in the BMDMs from HIV-Tg26 mice compared to those from WT mice. Similar to previous studies (Kavvadas et al., 2018), we speculate that Notch3 activation, in combination with HIV genes, in glomerular cells and BMDMs activates the migratory, proliferative and infiltrating properties of cells. We also speculate that intraperitoneal macrophages or cells of other lineages, such as regulatory T cells (FOXP3 positive), also have Notch3 activation in the HIV-Tg26 mice. Together, these changes might be responsible for the renal and systemic inflammatory responses, with N3KO leading to decreased systemic TNF and MCP-1, as well as reduced recruitment of inflammatory cells in the kidney and likely in skin lesions, thereby increasing the lifespan of the HIV-Tg-N3KO mice.

Because the Notch pathway is activated in glomerular diseases with or without HIV-1, it remains to be determined whether HIV-1 can induce the activation of Notch3 directly. Using a pseudotyped replication-defective HIV-1 construct, we found that HIV-1 can activate Notch3 directly in podocytes. In addition, N3KO in HIV-Tg26 mice resulted in a significant drop in the expression of *nef* and *env*, and N3IC significantly increased the HIV-LTR promoter activity, indicating feedback regulation of N3IC and HIV-1 genes. In support of these findings, the HIV Tat protein was shown to interact with the extracellular domain of the Notch receptors and was suggested as a non-canonical ligand for Notch activation (Shoham et al., 2003; Guo et al., 2018; Sakamoto et al., 2005; Fan et al., 2016). In addition, previous studies in HIV-Tg26 mice and human podocytes showed that HIV-1 can precipitate the development of HIVAN and affect the cytoskeletal structure of podocytes cultured from children with HIVAN (Tang et al., 2020; Xie et al., 2014). Moreover, there are two RBP-JK binding sites adjacent to the NFκB-binding sites on the HIV-LTR promoter. RBP-JK binds to the LTR and promotes recruitment of histone deacetylate (HDAC) and polycomb repressive complexes (PRCs) in CD4^+^ T cells. Knockdown of RBP-JK or PRC was shown to result in proviral activation (Mbonye and Karn, 2014; Tyagi and Karn, 2007; Sharma et al., 2020). RBP-JK is a known transcriptional repressor, which changes into a transcriptional activator upon NIC binding. Thus, we speculate that NIC binding to RBP-JK in HIV-Tg26 mice leads to activation of pathways downstream of Notch3. Further studies will be required to clarify this mechanism, because, in HIV-Tg26 mice, the LTR promoter is primarily bound to the active p50/p65 heterodimer (Bruggeman et al., 2001). Moreover, it should be noted that the viral LTR in HIV-Tg26 mice is largely dependent on the NFκB sites because Tat functions are inefficient, leading to the inability of murine cyclin T1 to form a complex with positive transcription elongation factor b (pTEFb). Thus, any direct effects of Notch3 on HIV gene expression might be more dramatic in mouse models. Here, we show that, in human podocyte lines, the LTR is activated by N3IC, and N3IC is induced by the HIV construct, revealing that this mechanism exists in human, although further investigations are required.

Our study showed decreased HIV gene expression as well as decreased inflammation upon N3KO, indicating that the reduction in inflammation is a combination of direct effects by N3KO and decreased HIV gene expression. Thus, N3KO might exert dual effects in HIV-1. Further, Notch3 inhibition appears to be an excellent strategy to reduce inflammation and HIV gene expression, although we stress that a combined approach to decrease the activity of both Notch3 and Notch4 could have better outcomes. Age-related vascular lesions have been detected in Notch3 mice (Henshall et al., 2015; Ragot et al., 2016); therefore, targeted delivery of a Notch3 inhibitor might be beneficial.

We have found that the Notch3 pathway is activated in BMDMs and several kidney cell types from HIV-Tg26 mice. Activation of the Notch3 pathway leads to significant systemic and renal inflammatory changes that precipitate the development of CKD and affect the survival of these mice. Our data also suggest that HIV-1 genes can directly activate the Notch3 pathway in immune cells and human podocytes. It is also tempting to speculate that the Notch3 pathway interacts with other cells of the innate immune system, as well as major risk factors that predispose to the development of CKDs in people of African descent, including the APOL1 risk variants. Nonetheless, regardless of the APOL1 risk variants, it appears that new therapies that target the NOTCH3 pathway could have a key role in preventing the development of HIV-related CKD by decreasing HIV-1 genes and inflammation in kidneys and likely other organs.

## MATERIALS AND METHODS

### Sex as a biological variable

Human biopsy samples were unidentified, age matched, and obtained from both males and females. In mouse studies, males and females were included in equal numbers, and no difference between the sexes was apparent.

### Human tissues

Renal biopsies were from unidentified patients with HIVAN, and age-matched unaffected controls. All patients had nephrotic-range proteinuria, and ART had not been started. The use of these tissues for the study was evaluated and approved for use by the Human Protection Program at the University of Kansas Medical Center and University of Feinstein Institute for Medical Research, Zucker School of Medicine at Hofstra-Northwell. All clinical investigations were performed according to the principles expressed in the Declaration of Helsinki.

### Animal care and studies

All experiments were performed under the guidelines of the Guide for the Care and Use of Laboratory Animals of the National Institutes of Health (NIH), as approved by the Institutional Animal Care and Use Committee of the University of Kansas Medical Center (Kansas City, KS, USA). Mice were housed in micro-isolator cages on a high-efficiency particulate air-filtered, ventilated rack under aseptic and pathogen-free conditions. The heterozygous HIV-Tg26 mice show all characteristics of HIVAN and were a kind gift from Dr Paul Klotman (Baylor College of Medicine, Houston, TX, USA) (Kopp et al., 1992). Notch3^d1^ (N3KO) mice were obtained from The Jackson Laboratory (JAX:023807). All mice were maintained on FVB/N background. Sample size was based on power analysis and previous studies (Puri et al., 2019). All experiments were performed/reported under Animal Research: Reporting of *In Vivo* Experiments (ARRIVE) guidelines (Kilkenny et al., 2010) and according to the Guide for the Care and Use of Laboratory Animals of the NIH, as approved by the Institutional Animal Care and Use Committee of the University of Kansas Medical Center. Mice were housed in micro-isolator cages on a high-efficiency particulate air-filtered, ventilated rack. Conditions were aseptic and pathogen free. Comparisons were made between WT (normal control), HIV-Tg26 (positive control), N3KO mice (another control) and HIV-Tg-N3KO mice (new genotyping to be tested) at 3 months of age. N3KO mice (B6;129S1) were backcrossed onto FVB/N background for at least six generations to be congenic before breeding with HIV-Tg26 mice. Before euthanasia at 3 months of age, overnight urine was collected in metabolic cages for proteinuria and albumin-to-creatinine ratio evaluation. Blood was collected at the time of euthanasia for serum isolation. One half of a kidney was stored in paraformaldehyde overnight and changed to 70% ethanol. The other half and second kidney were snap frozen for RNA isolation and protein lysates and stored at −80°C immediately. Another set of mice was monitored for 6 months for mortality studies.

### Antibodies and reagents

The following antibodies were used: anti-β-actin (1:1000; Sigma-Aldrich, St Louis, MO, USA; A5441); anti-MMP10 (1:1000; My Bio Source, San Diego, CA, USA; MBS2027749); anti-Notch3, anti-Dll4 and anti-jagged 1 (1:1000; Abcam, Cambridge, UK; ab23426, ab7280 and ab7771, respectively); anti-CD68 for detection in mice (1:100; Abcam, ab125212); anti-CD68 for detection in human (1:100; BioCare Medical, CM033A); anti-p-NFκB (Cell Signaling Technology, 3033s); Mouse Immune Cell Phenotyping IHC Antibody Kit (Cell Signaling Technology, 37495; containing anti-FOXP3, anti-CD11c, anti-CD8 and anti-granzyme B antibodies); secondary antibodies goat anti-mouse IgG H&L (Alexa Fluor^®^ 594; Abcam, ab150116) and goat anti-rabbit IgG H&L (Alexa Fluor^®^ 488; Abcam, ab150077).

### Renal function evaluation

BUN levels were quantified using a Quantichrom Urea Assay Kit (Bioassay Systems, Hayward, CA, USA) according to the manufacturer's protocol. Proteinuria was measured in 2 µl urine, which was loaded onto a 10% SDS-polyacrylamide gel for electrophoresis, followed by Coomassie Blue staining. Bovine serum albumin (BSA) (2 µl; 10 mg/ml) was used as a positive control. Albumin in urine was assessed by an enzyme-linked immunosorbent assay (ELISA) kit from Bethyl Laboratories (Houston, TX, USA). Creatinine levels in the same urine samples were quantified using a QuantiChrome Creatinine Assay Kit (Bioassay Systems).

### Histology

Kidneys were fixed in 4% paraformaldehyde overnight and transferred to 70% ethanol. The tissues were processed and embedded in paraffin at the core facilities at the University of Kansas Medical Center. Five-micrometer sections were stained with Hematoxylin and Eosin, periodic acid–Schiff (PAS) and Mason trichrome. Slides were examined unaware of experimental group and scored for tubulointerstitial disease and glomerular injury (*n*=6, each group) as described previously (Puri et al., 2019). Briefly, the area was inspected, and percentage of tubulointerstitial disease (enlarged, reactive tubular nuclei, tubular casts, tubular dilation and interstitial fibrosis) was estimated. The number of glomeruli with visible disease (segmental and global sclerosis, collapsed phenotype, adhesion to Bowman capsule) was counted, and the percentage of glomeruli involved was calculated for glomerular injury score. Percentage immune cell infiltration was calculated by estimating the percentage area of affected cortex.

### Cell culture

All cell lines used were free of mycoplasma contamination, as verified by a MycoAlert^®^ Mycoplasma Detection Kit (Lonza, Basel, Switzerland; LT07-118). Human immortal podocytes were a kind gift from Dr Moin Saleem (University of Bristol, Bristol, UK) (Saleem et al., 2002). Immortal podocytes were maintained in a growth medium containing RPMI 1640 (Hyclone, Thermo Fisher Scientific, Waltham, MA, USA) [supplemented with 10% fetal bovine serum, 1× penicillin/streptomycin, 1× insulin-transferrin-selenium (GenDEPOT, Katy, TX, USA)] at 33°C for SV40T antigen expression. To inactivate SV40T antigen, cells at 50% confluency were moved to 37°C in 5% CO_2_ and allowed to differentiate for 7-10 days.

#### Podocyte transductions

To study the induction of Notch3 by HIV-1, pNL4-3:ΔG/P-GFP construct was used. Generation of this construct has been described previously (Chandel et al., 2015; Husain et al., 2002). Briefly, in HIV-1 proviral construct (pNL4-3) the *gag*/*pol* regions were substituted with green fluorescence protein (GFP) reporter gene. As a negative control (WT), pHR-CMV-IRES2-GFP-ΔB construct containing HIV-LTR and GFP empty expression vector was used. These constructs were pseudotyped with VSV.G envelope separately to generate viral particles in 293T cells. Viral particles were used to infect differentiated conditionally immortalized podocytes for 2 days as described (Puri et al., 2019). Podocytes were lysed using RIPA buffer [137 mM NaCl, 50 mM Tris-HCl pH 7.5, 12 mM EDTA, 1% IGEPAL and complete protease inhibitor (Thermo Fisher Scientific, St Louis, MO, USA)] and stored at −80°C until use.

#### Luciferase promoter reporter assays

The HIV-LTR promoter reporter luciferase construct has been described previously and was a kind gift from Dr Wang (Li et al., 2017). N3IC-expressing construct was developed as previously reported (Wang et al., 2007). Co-transfections were carried out with empty vector (PCDNA3.1), N3IC construct (PCDNA3.1 N3IC) and HIV-LTR (pGL3-LTR-Luc) using Lipofectamine™ LTX (Thermo Fisher Scientific). Twenty-four hours after transfections, cells were lysed with passive lysis buffer, and a Dual Luciferase Assay Kit (Promega, Madison, WI, USA) was used to assess luciferase activity using a luminometer (Lumat LB 9507, Berthold Technologies, Bad Wildbad, Germany). Reporter activity was normalized to renilla luciferase activity and expressed as fold change in relative light units.

#### Isolation and culture of BMDMs

Mice were euthanized, and legs were removed under aseptic conditions. Legs were deskinned, and tibiae and femurs were separated. Both ends of the tibia and femur were cut under sterile conditions, and marrow was flushed by placing femur or tibia on a cut pipette tip placed into a 1.5 ml tube. The tubes were then spun down at 250 ***g*** for 5 min at room temperature to pellet cells. The cell pellet was resuspended in tissue culture plate with BMDM medium containing Iscove's modified Dulbecco's medium (IMDM) supplemented with glutamine, 1% minimum essential medium non-essential amino acids, 1% sodium pyruvate, 1% penicillin/streptomycin, 0.05 mM β-mercaptoethanol and 30% L929 supernatant for a source of macrophage colony stimulating factor (see ‘L929 medium’ section). Cells were incubated for 7 days at 37°C in 5% CO_2_ with addition of 5 ml medium/10 cm^2^ dish at day 3. On day 8, the cells were scraped gently in PBS, resuspended and counted. Cells (2×10^6^) were plated in 10 cm^2^ dishes for 3 days and lysed. Lysates were stored in −80°C until use.

#### L929 medium

L929 cells were grown to confluency in IMDM containing 10% fetal bovine serum, 1% non-essential amino acids, sodium pyruvate and 1× penicillin/streptomycin. After 3 days, medium was collected, filtered, sterilized and stored at −80°C until use.

### Immunolabeling

Sections from kidneys of WT, N3KO, HIV-Tg26 and Tg-N3KO mice were deparaffinized with xylene and hydrated in ethanol. These sections were then boiled in sodium citrate buffer (10 mM sodium citrate, 0.05% Tween 20, pH 6.0) and cooled to room temperature. To block endogenous peroxidase activity, sections were incubated for 30 min with 3% hydrogen peroxide. They were then washed in PBS and blocked with 10% normal serum (from the species the secondary antibody was raised in, in PBS) for 1 h, followed by a 1 h incubation in a humidified chamber with primary antibodies. Sections were washed three times with PBS, then incubated for 1 h in 1:400 diluted biotin-conjugated secondary antibodies (Vector Laboratories) for immunohistochemistry and fluorescein/Texas Red-conjugated antibodies for immunofluorescence, followed by another wash. For immunofluorescence, the slides were mounted using Alexa Fluor^®^ 594 or Alexa Fluor^®^ 488 with DAPI (Abcam). For immunohistochemical analysis, the slides were further incubated with avidin-biotin-peroxidase complex (ABC Elite; Vector Laboratories), which was detected with diaminobenzidine (DAB; Sigma-Aldrich). Some kidneys were counterstained with Hematoxylin. Tissue sections were then dehydrated with graded ethanols and mounted with permount (Fisher Scientific, Pittsburgh, PA, USA). Slides were viewed on a DMLB 100s upright microscope (Leica, Wetzlar, Germany).

### Quantification of histology and immunolabeling

For counting CD68^+^, FOXP3^+^ and CD8^+^ cells, images were captured from all areas in which these cells were present (*n*=5-6 per kidney, six kidneys per group). Cells positive for Hematoxylin (total cells in the section) and cells with DAB staining (only positive for CD68, CD8 or FOXP3) were counted, and the percentages of positively labeled cells were calculated per section and averaged. For quantification of immunofluorescence intensity, ImageJ was used, and percentage intensity was measured from each image and averaged. For counting Notch3 expression, two to three images per kidney (from the glomerular region) were taken. For quantification of Notch3 intensity, vascular Notch3 values were subtracted from total intensity to assess glomerular and tubular labeling individually.

### Western blotting

Protein estimation was performed in cell lysates by a BCA protein assay kit (Bio-Rad, Hercules, CA, USA). For western blots, 75 μg protein was solubilized in 4× NuPage (Novex, Thermo Fisher Scientific) sample buffer [containing 25% Tris (2-carboxyethyl) phosphine (TCEP)]. Samples were heated to 65°C for 10 min before electrophoresis on 10% SDS-polyacrylamide gels. Samples were then transferred to polyvinylidene difluoride membranes. For blocking of immunoblots for 1 h at room temperature, 5% non-fat dry milk in Tris-buffered saline containing 0.1% Tween 20 (TBST) was used. After washing blots with TBST, overnight incubation was performed using the appropriate dilutions of primary antibodies. Blots were washed three times with TBST, then incubated for 1 h at room temperature in secondary antibodies (Vector Laboratories; 1:5000 dilution in blocking solution). Chemiluminescence was then used for detection (Western Lightning Plus ECL, Perkin Elmer, Waltham, MA, USA).

### RNA-seq

RNA-seq was carried out in kidneys from 3-month-old WT (*n*=3), HIV-Tg26 (*n*=7), N3KO (*n*=4) and HIV-Tg-N3KO (*n*=6) mice. Briefly, mouse kidneys were lysed in Trizol (Thermo Fisher Scientific) according to the manufacturer's protocol. Verification of RNA integrity, global transcriptomic analysis, preparation of RNA-seq libraries and sequencing of complementary DNA (cDNA) libraries were performed using Affymetrix Clariom D (Thermo Fisher Scientific) array at the Genomics Core Facility of the University of Kansas Medical Center as described previously (Chakravarthi et al., 2020; Sharma et al., 2021). A NovaSeq 6000 sequencing machine (Illumina, San Diego, CA, USA) was used to perform RNA-seq at a strand-specific 100-cycle paired-end resolution. The read quality was assessed using FastQC software. On average, the per sequence quality score measured in the Phred quality scale was above 30 for all samples. The sequenced reads were mapped to the combined mouse (GRCm38.rel97) and HIV-1 (GCF_000864765.1) genomes using STAR software (version 2.6.1c) (Dobin et al., 2013). On average, 95% of the sequenced reads mapped to the genome, resulting in between 27 and 32[Supplementary-material sup1] million mapped reads per sample, of which, on average, 86% were uniquely mapped reads. Transcript abundance estimates were calculated using the featureCounts software (Liao et al., 2014). Expression normalization and differential gene expression calculations were performed in DESeq2 software (Love et al., 2014) to identify statistically significant differentially expressed genes. *P*-values were adjusted for multiple hypotheses testing by the Benjamini and Hochberg method (Benjamini and Hochberg, 1995), establishing a false discovery rate for each gene. The resulting data were used to generate heatmaps and volcano plots for visualization.

### qPCR

Total RNA was isolated from tissues using Trizol (Thermo Fisher Scientific), following the manufacturer's protocol. Nanodrop analysis was used to determine RNA concentration and purity. Using a High-Capacity Reverse Transcription Kit (Thermo Fisher Scientific), cDNA was prepared from 2 µg total RNA. qPCR was performed using Power SYBR Green Master Mix (Applied Biosystems, Waltham, MA, USA). Results were normalized to 18S ribosomal RNA (rRNA) expression and calculated using comparative ΔΔCT. Ct values for each gene were obtained. By subtracting the housekeeping gene value from the Ct value of the samples, delta Ct (ΔCT) values were calculated. Using the ΔCt values of the WT controls (*n*=3), an average was found. The average ΔCt value of the WT controls was then subtracted from the ΔCt values of all samples to obtain a ΔΔCt value. To calculate relative fold change, the 2-ΔΔCt formula was used for each control or gene of interest value. Values from each group were averaged and shown as a relative fold change in gene expression as previously shown. The primers used in this study are listed in Table S3.

### ELISA and chemokine/cytokine profiling

Serum was assessed for the presence of TNF and MCP-1 using mouse ELISA kits (R&D Systems, Minneapolis, MN, USA) according to the manufacturer's instructions. For analysis of chemokines and cytokines in serum of HIV-Tg26 and Tg-N3KO mice, serum samples from three male mice (200[Supplementary-material sup1]μl each) were pooled from each group. From each group, 150 ml serum was used for chemokine array using a Proteome Profiler Mouse Chemokine Array Kit (R&D Systems; ARY020), and 200[Supplementary-material sup1]μl was used from each group for cytokine array using a Proteome Profiler Mouse Cytokine Array Kit (R&D Systems; ARY028). The manufacturer's instructions were followed.

### Statistics

Data are plotted as mean±s.e. Unpaired two-tailed Student's *t*-test was used to determine the statistical significance of differences between control and test groups. To compare more than two groups, a one-way ANOVA followed by Tukey's multiple comparison test was performed, using GraphPad Prism. *P*<0.05 was considered statistically significant.

## Supplementary Material

10.1242/dmm.052056_sup1 Supplementary information
