# Supplementary material for: Notch3 deletion regulates HIV-1 gene expression and systemic inflammation to ameliorate chronic kidney disease
Source: Dis Model Mech. 2025 Feb 25;18(2):DMM052056. doi: 10.1242/dmm.052056 (PMC11892680; doi:10.1242/dmm.052056)
Supplement: Supplementary information [file dmm-18-052056-s1.pdf]

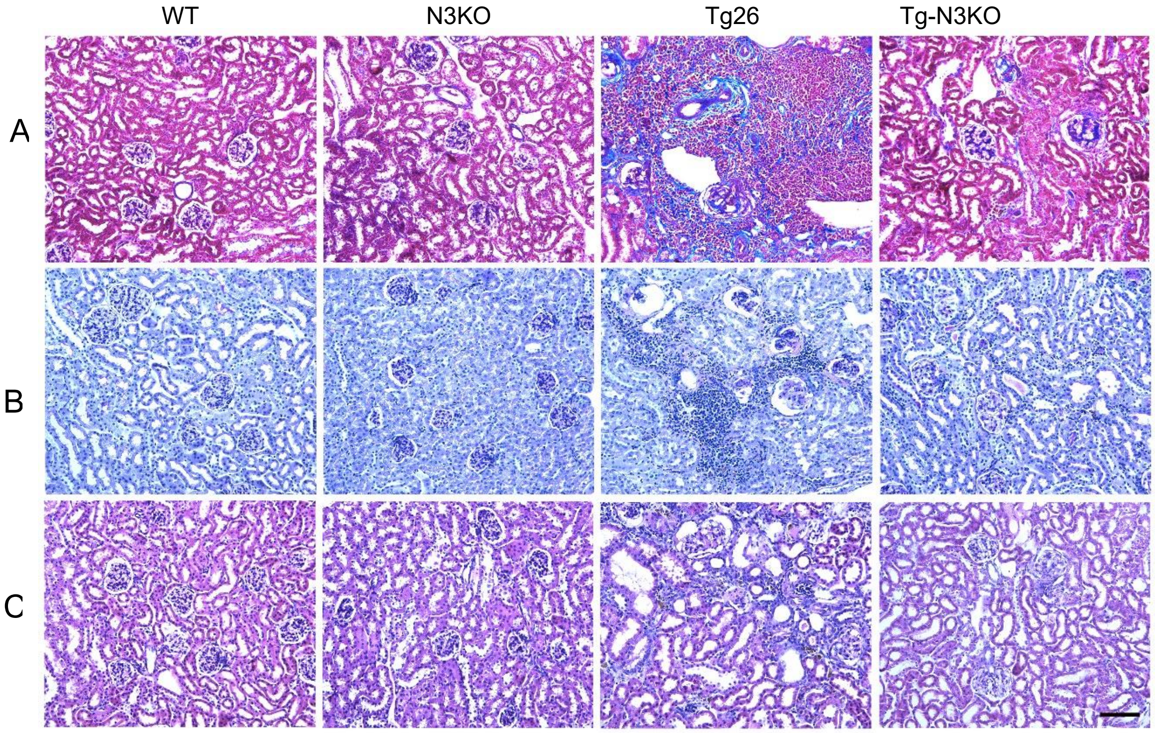

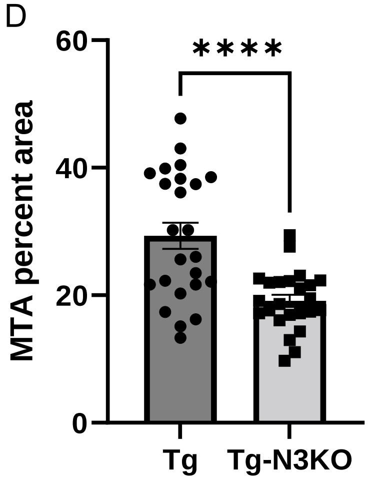

Fig S1. Notch3 deletion ameliorates histological lesions in Tg26 mice: (A,B,C) Kidney sections from 3 months old female WT, N3KO, HIV-Tg26 and HIV-Tg-N3KO mice were stained with Mason Trichrome (MTA), Periodic acid Schiff (PAS) and Hematoxylin & Eosin (H&E) respectively. Representative image shown from each group. Note massive glomerulosclerosis, fibrosis, tubulointerstitial injury and infiltration in Tg26 sections which was reduced in HIV-Tg-N3KO sections. (D) quantification of MTA represented as percent area stained for MTA. (scale bar 50μm), kidneys (****P*<0.0001).

Disease Models & Mechanisms • Supplementary information

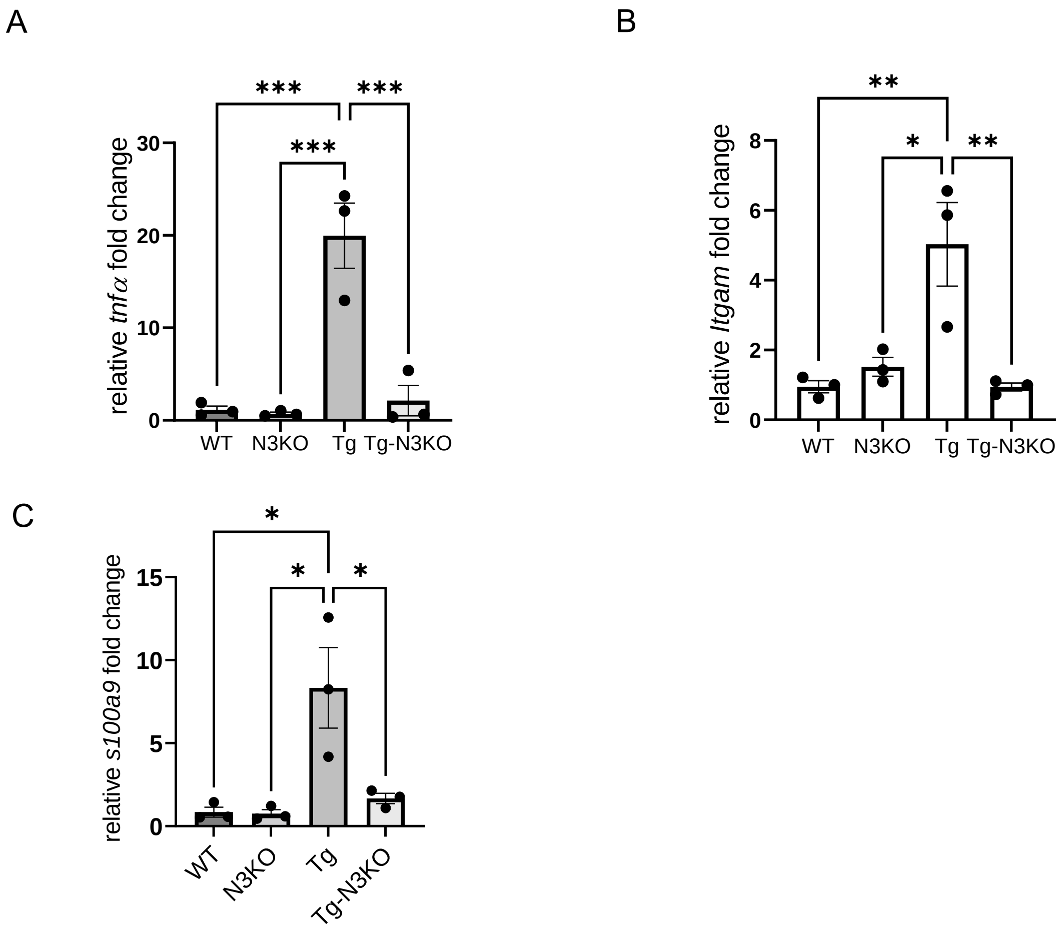

Fig. S2. Quantitation of major genes downregulated in Notch3 deleted Tg26 mice. (A, B, and C) Quantitative PCR validating the upregulated expression of *tnfa*, *itgam* and *s100a9*, genes related to macrophage associated inflammation in Tg26 mice that almost normalized in Tg-N3KO kidneys (**P*<0.05, ** *P*<0.01, *** *P*<0.001).

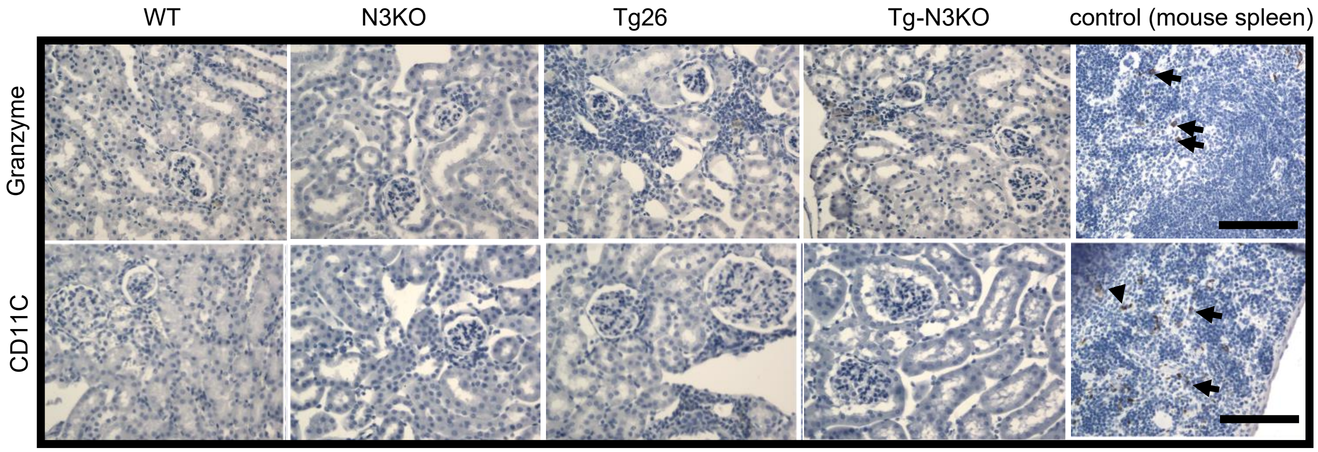

Fig. S3. Effects of Notch3 deletion on Granzyme and CD11C in kidneys of Tg26 mice. Immunohistochemistry was performed in paraffin sections from WT, N3KO, Tg26 and Tg-N3KO kidneys for presence of Granzyme and CD11C. Data is representative of 4-5 kidneys from each group of male and female mice. Control represents spleen of a normal mouse where positive labeling is noticed (arrows). Scale bar: 100μm.

A

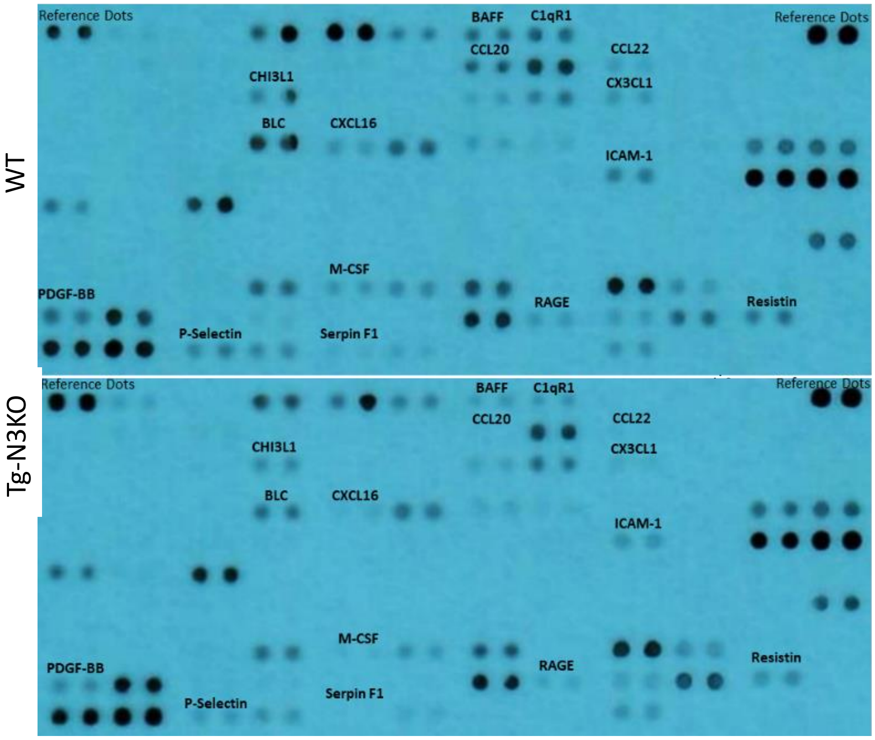

B

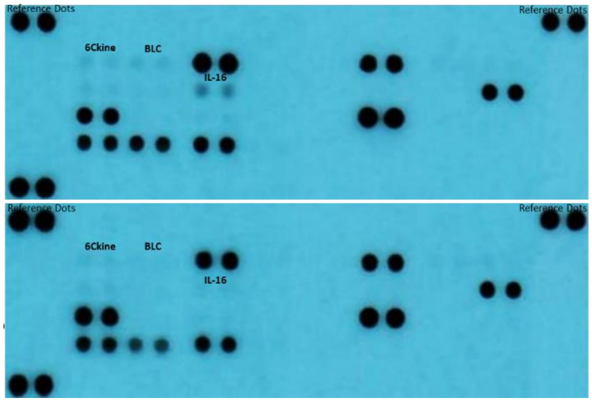

Fig. S4. Cytokine and chemokine regulation by Notch3. (A and B) Dot blots comparing the expression of various cytokines (A) and chemokines (B) in the pooled serum from Tg26 (n=3) and Tg-N3KO (n=3) mice (males). Dots in duplicates represent one analyte. Dots having ≥ 1.5-fold differences in mean pixel density between Tg26 and N3-TgKO are labelled.

Disease Models & Mechanisms • Supplementary information

Table S1. List of differentially expressed cytokines.

Name of Analytes	Tg vs Tg-N3KO
Angiopoietin-1	-1.14
Angiopoietin-2	-1.34
BAFF/BLyS/TNFSF13B	-1.61
C1q R1/CD93	-1.92
CCL20/MIP-3 alpha	-6.61
CCL22/MDC	-3.53
CD14	-1.38
Chitinase 3-like 1	-1.65
CX3CL1/Fractalkine	-1.58
CXCL13/BLC/BCA-1	-1.83
CXCL16	-3.27
ICAM-1/CD54	-1.46
M-CSF	-8.75
PDGF-BB	-1.48
Periostin/OSF-2	-1.45
RAGE	-1.90
Resistin	-1.71
P-Selectin/CD62P	-1.97
Serpin E1/PAI-1	-1.26
Thrombopoietin	-1.58
DPPIV/CD26	-1.72
Endoglin/CD105	-4.60
VEGF	-1.37

WISP-1/CCN4	-2.20
Serpin F1/PEDF	-2.28
CCL6/C10	+1.91
Myeloperoxidase	+1.04
Osteopontin (OPN)	+1.25
Osteoprotegerin/ TNFRSF11B	+1.10
Pentraxin 2/SAP	+1.30
RBP4	+1.12
Reg3G	+1.62

Table S1 showing the list of cytokines altered in the serum of Tg-N3KO mice compared to Tg26 mice (samples pooled from n=3, each group).

Table S2. List of differentially expressed chemokines.

Name of Analyte	Tg vs Tg-N3KO
6Ckine/CCL21/SLC/Exodus-2	-1.69
BLC/CXCL13/BCA-1	-2.08
C10/CCL6/MRP-1	-1.36
Chemerin/RARRES2	-1.10
CTACK/CCL27/ALP/ILC/Eskine	-1.23
Fractalkine/CX3CL1	-2.04
IL-16	-3.65
JE/CCL2/MCP1	-1.30
LIX/GCP-2/ENA-78	-1.06
MCP-5/CCL12	-1.24
MDC/CCL22/ABCD-1	-1.12
MIG/CXCL9/CRG-10/CMK	-1.07
RANTES/CCL5/SISd	-1.33
CXCL16/SRPSOX	+1.43
Eotaxin/CCL11	+1.14
MCP2/CCl8/HC14	+1.21

Table S2 showing the list of chemokines altered in the serum of Tg-N3KO mice compared to Tg26 mice (samples pooled from 3 mice in each group).

Table S3. List of primers used in the study.

Gene	Forward primer (5'-3')	Reverse primer (5'-3')
mmp10	GCCCAGCTAACTTCCACCTTT	GAGAGTGTGGATCCCCTTTGG
ccl2	TAAAAACCTGGATCGGAACCAAA	GCATTAGCTTCAGATTTACGGGT
chil3	CAGCATATGGGCATACCTTT	CAGACCTCAGTGGCTCCTT
retnlg	AGGAACTTCTTGCCAATCG	GCCTGAAGCCGTGATACT
s100a9	CAGCATCATACACTCCTCAAAG	AATGGTGGAAGCACAGTT
itgam	AAACCACAGTCCCGCAGAGA	CGTGTTCACCAGCTGGCTTA
ubd	CCAGATCCTTCTGCTAGACTCC	ACTCCACCAGAAACAAGGGCAG
il6	TAGTCCTTCCTACCCCAATTTCC	TTGGTCCTTAGCCACTCCTTC
Rn18s	GCAATTATTCCCCATGAACG	GGCCTCACTAAACCATCCAA
Tnfa	ACCCTCACACACTCAGATCATCTTC	TGGTGGTTTGCTACGACGT
Nef	ATGGGTGGCAAGTGGTCAA	TCAGCAGTTCTTGAAGTACTC
Env	TGTGTAAAATTAACCCCACTCTG	ACAACTTATCAACCTATAGCTGGT

Disease Models & Mechanisms • Supplementary information
